# CT angiography vs echocardiography for detection of cardiac thrombi in ischemic stroke: a systematic review and meta-analysis

**DOI:** 10.1007/s00415-020-09766-8

**Published:** 2020-03-05

**Authors:** Nina-Suzanne Groeneveld, Valeria Guglielmi, Mariska M. G. Leeflang, S. Matthijs Boekholdt, R. Nils Planken, Yvo B. W. E. M. Roos, Charles B. L. M. Majoie, Jonathan M. Coutinho

**Affiliations:** 1 Department of Neurology, Amsterdam UMC, University of Amsterdam, Amsterdam, The Netherlands; 2 Department of Clinical Epidemiology and Biostatistics and Bioinformatics Amsterdam Public Health, Amsterdam UMC, University of Amsterdam, Amsterdam, The Netherlands; 3 Department of Cardiology, Amsterdam UMC, University of Amsterdam, Amsterdam, The Netherlands; 4 Department of Radiology and Nuclear Medicine, Amsterdam UMC, University of Amsterdam, Amsterdam, The Netherlands

**Keywords:** Ischemic stroke, Cardiac emboli, Cardiac computed tomography, Echocardiography, Thrombus

## Abstract

**Background and purpose:**

Cardiac thrombi are an important cause of embolic stroke. We studied the diagnostic yield and diagnostic accuracy of cardiac CT angiography (CTA) compared to echocardiography for detection of cardiac thrombi in ischemic stroke patients.

**Methods:**

We performed a systematic review and meta-analysis of the literature on cardiac CTA versus echocardiography for detection of cardiac thrombi in ischemic stroke patients. We included studies (*N* ≥ 20) in which both cardiac CTA (index test) and echocardiography (reference test) were performed and data on cardiac thrombi were reported. Results were stratified for type of echocardiography: transesophageal (TEE) vs transthoracic (TTE).

**Results:**

Out of 1530 studies, 14 were included (all single center cohort studies), with data on 1568 patients. Mean age varied between 52 and 69 years per study and 66% were men. Reported time intervals ranged from 0 to 21 days between stroke and first test, and from 0 to 199 days between tests. In ten studies that compared CTA to TEE, CTA detected cardiac thrombi in 87/1385 (6.3%) patients versus 68/1385 (4.9%) on TEE (*p* < 0.001). In four studies comparing CTA to TTE, CTA detected thrombi in 23/183 (12.5%) patients versus 12/183 (6.6%) on TTE (*p* = 0.010). Pooled sensitivity and specificity of CTA versus TEE were 86.0% (95% CI 65.6–95.2) and 97.4% (95% CI 95.0–98.7), respectively.

**Conclusions:**

CTA may be a promising alternative to echocardiography for detection of cardiac thrombi in patients with ischemic stroke, especially now that CTA is standard care for patient selection for endovascular treatment. However, studies were too heterogeneous and of insufficient methodological quality to draw firm conclusions. Large, prospective studies on this topic are warranted.

**Electronic supplementary material:**

The online version of this article (10.1007/s00415-020-09766-8) contains supplementary material, which is available to authorized users.

## Introduction

Thromboembolism from a cardiac source accounts for up to one-third of all ischemic strokes [1–3]. Establishing cardioembolism is important in patients with ischemic stroke, since these patients are commonly managed with anticoagulation instead of antiplatelet therapy [4]. Thrombi in the left atrial appendage (LAA) or left atrium (LA) secondary to atrial fibrillation (AF) are a major cause of cardioembolic stroke [4]. However, AF is often paroxysmal, which can be challenging to detect [5]. Moreover, conditions other than AF can also cause cardiac thrombi, such as thrombi in the left ventricle after myocardial infarction [4]. Timely detection of cardiac thrombi allows for early optimization of medical therapy.

Transthoracic echocardiography (TTE) and transesophageal echocardiography (TEE) are the first-line cardiac imaging modalities in stroke patients [6, 7]. Advantages are portable equipment, wide availability and relative inexpensiveness. TEE is more sensitive than TTE for detection of thrombi in LAA and LA [6, 7]. However, TEE is time-consuming, burdensome for the patient, may require sedation, and has a low—but not negligible—risk of esophageal complications [6]. TTE is more suitable for evaluation of the left ventricle [7], but may be difficult in patients with poor acoustic windows, with obesity or breast implants, and in those suffering from chronic obstructive pulmonary disease. The quality of echocardiography is also dependent on operator expertise. Cardiac CT angiography (CTA) is an alternative imaging technique for detection of cardiac thrombi. It may be a particularly useful modality in acute ischemic stroke patients, now that CTA of the cervical and intracranial arteries has become routine care to determine eligibility for endovascular treatment and cardiac CTA could be included in the same imaging protocol [8–10]. Furthermore, the additional radiation associated with cardiac CTA is low with modern CT scanners [11]. Our aim was to study the diagnostic yield and diagnostic accuracy of CTA for detection of cardiac thrombi in stroke patients, compared to echocardiography, by performing a systematic review and meta-analysis of the literature.

## Methods

### Study design

We searched Medline and Excerpta Medica database (EMBASE) from inception to June 7th 2019 using the following search terms: ischemic stroke, cardiac CT angiography, and cardiac thrombus (Online Resource Table I). Rayyan (https://rayyan.qcri.org) was used for study screening [12]. Title and abstract screening was performed independently by two authors (NSG and VG). Full-length review of potentially relevant studies and conference abstracts was performed by one author (NSG). We also screened reference lists of included articles to identify additional potentially relevant studies. Final decisions regarding study selection were reached through consensus. We registered the protocol in the International Prospective Register of Systematic Reviews (PROSPERO), IC CRD42018103658. We wrote our report according to Preferred Reporting Items for Systematic Reviews and Meta-Analyses (PRISMA) guidelines for Diagnostic Test Accuracy (DTA) studies [13].

### Inclusion and exclusion criteria

We included studies in any language that: (1) performed cardiac CTA as well as echocardiography (TEE or TTE); (2) in patients with ischemic stroke or transient ischemic attack (TIA); (3) allowed data extraction on cases with cardiac thrombi; (4) had a randomized controlled trial or a cross-sectional diagnostic accuracy study design (cohort or case control with a minimum population size of 20 patients). Abstracts containing sufficient data were also included. In case of overlapping patient cohorts between studies, the study with the largest sample size was included.

### Index test and reference standard, study endpoints

A true gold standard for detection of cardiac thrombi is lacking. In our study, cardiac CTA was considered the index test and echocardiography the reference standard. The decision to use echocardiography as the reference test was based on the fact that international guidelines recommend echocardiography as first-line screening method for imaging of the heart in ischemic stroke patients [6, 7]. Results were stratified for type of echocardiography: CTA versus TEE and CTA versus TTE. Studies in which patients underwent both TTE and TEE, but only TEE results were clearly reported, were considered as CTA versus TEE. The primary endpoint was diagnostic yield, defined as proportion of patients with a cardiac thrombus on CTA or echocardiography. The secondary endpoint was diagnostic accuracy, defined as sensitivity and specificity of cardiac CTA compared to echocardiography. Due to the lack of a true gold standard, diagnostic yield and not diagnostic accuracy was chosen as primary endpoint.

### Quality assessment and data extraction

Both quality assessment and data extraction were independently performed by two authors (N.S.G. and V.G.). In situations of disagreement, a third author made the final decision (M.M.G.L. for quality assessment and J.M.C for data extraction). The Quality Assessment of Diagnostic Accuracy Studies (QUADAS)-2 checklist was used to assess risk of bias and concerns regarding applicability [14]. The different components of the QUADAS-2 guidelines were tailored to our research question (Online Resource Tables IIa–IIb).

We extracted data on study characteristics (study design, publication year, sample size) and study population characteristics (mean age, sex, ischemic stroke or TIA, presence or absence of cardiac medical history/disease). We also collected data on CTA and echocardiography techniques, such as type of scanner, ultrasound frequency, cardiac imaging protocols, definition of cardiac thrombus, order of testing, time interval between stroke onset and first test, time interval between tests, and amount of cardiac thrombi found through cardiac CTA and echocardiography. We collected data on thrombi detected in any location in the heart (LAA, LA, left ventricle, right atrium, right ventricle).

### Statistical analysis

To determine differences in diagnostic yield, the OpenEpi^®^ software program version 3.01 (Open Source Epidemiologic Statistics for Public Health) was used to perform a McNemar test for paired data. Data of patients who underwent both CTA and TEE, and CTA and TTE, respectively, were analyzed using a two-sided McNemar test with binomial enumeration (Mid-P Exact) and a significance level of 0.05. To study diagnostic accuracy, sensitivity and specificity of CTA versus TEE and CTA versus TTE were calculated for all studies based on reported cardiac thrombus cases. Forest plots were generated using Review Manager version 5.3 (RevMan, Copenhagen: The Nordic Cochrane Centre, The Cochrane Collaboration, 2014). Studies for which both sensitivity and specificity were estimable were included in a meta-analysis. Summary receiver operating curves (SROC) with estimated area under the curve (AUC) and pooled sensitivity and specificity were generated using R version 3.2.5 (R Foundation for Statistical Computing, Vienna, Austria) using the Reitsma bivariate mixed effects model incorporated in the mada package version 0.5.8. Pooled sensitivity and specificity were also generated for a subgroup of studies that used 64-slice CT scanners or newer generations and for studies in which the reported time interval between CTA and echocardiography was approximately 1 day (mean/median/absolute amount of up to 1 day).

## Results

### Study selection

**Fig. 1:**
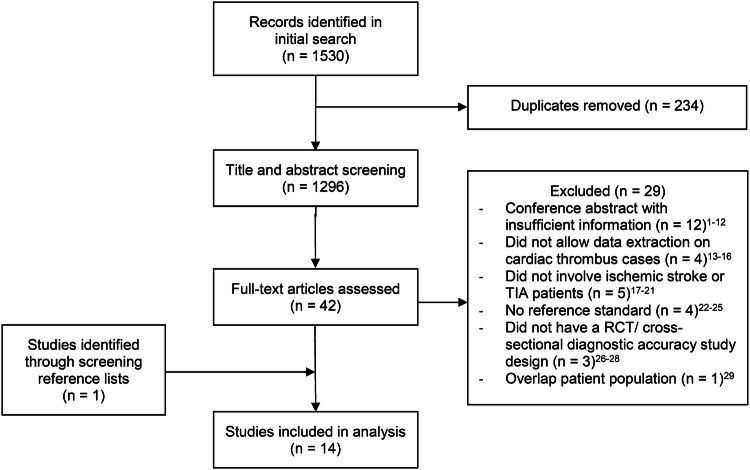
Flowchart of study selection**.** TIA indicates transient ischemic attack; RCT, randomized controlled trial. For detailed data of excluded studies please see Online Resource Reference list of excluded studies, appendix to Fig. 1

A flowchart of the study selection is provided in Fig. 1. We identified 1530 publications in the initial search, of which 42 were selected for full-length review. Of these, 14 articles fulfilled the inclusion criteria and were included in the analyses [8, 15–27].

### Study quality

Overall, the risk of bias was highest for the domains ‘patient selection’ and ‘reference standard’ (high or unclear risk of bias in 5/14 (35.7%) and 6/14 (42.9%) studies, respectively) (Online Resource Figure Ia and Ib). Regarding the domain ‘patient selection’, studies did not describe clearly why and/or how many patients were excluded. Regarding the domain ‘reference standard’, studies were unclear about whether the reference standard results were interpreted without knowledge of the results of the index test. In addition, for both the index and reference tests, there were issues with lack of reporting of the definition of a cardiac thrombus. Ten out of 14 (71.4%) studies defined cardiac thrombus on CTA and 8/14 (57.1%) on echocardiography. Only 2 out of 14 (14.3%) studies had a low risk of bias for all of the domains of the QUADAS-2 checklist [16, 18]. Concerns regarding applicability were generally low except for the domain ‘interpretation and conduct of the index-test’ for which two studies scored a high concern due to using outdated CT scanners (Imatron C-100) [15, 16].

### Study characteristics

The study characteristics of the 14 included studies are shown in Table 1. All studies were single center cohort studies (12 prospective and 2 retrospective), published between 1989 and 2017. In total, data of 1568 patients were available. Sample sizes of individual studies ranged from 20 to 374 patients.

**Table 1:** Study design and patient characteristics

References	Study design	Number of patients	Mean age in years (SD or range)	Men (%)	Population characteristics | proportion of patients with AF (%)
Helgason [15]	Prospective cohort	38	61 (18–86)	NR	Stroke of suspected cardioembolic source | 7/38 (18.4)
Love [16]	Prospective cohort	36	60 (NR)	20 (56)	Stroke or TIA of suspected cardioembolic source | NR
Hur [17]	Retrospective cohort	101	69 (45–81)	63 (62)	Stroke of suspected cardioembolic source | 21/101 (20.8)
Hur [18]	Prospective cohort	137	61 (13)	95 (69)	Stroke of suspected cardioembolic source | 57/137 (41.6)
Boussel [19]	Prospective open-pilot	39	63 (11)	38 (83)	Stroke | 3/39 (7.7)
Kim [20]	Prospective cohort	314	65 (13)	186 (59)	Stroke of suspected cardioembolic source | 72/314 (22.9)
Ko [21]	Prospective cohort	75*	67 (58–72)	42 (56)	Stroke | 48/143 (33.6)
Hur [22]	Prospective comparison	83	63 (36–83)	56 (67)	Stroke of suspected cardioembolic source | 49/83 (59.0)
Kim [23]	Prospective cohort	62*	68 (60–74)	46 (74)	Stroke without AF and without cardiac disease | 0/62 (0.0)
Sipola [24]	Prospective cohort	140	60 (10)	95 (68)	Stroke or TIA of suspected cardioembolic source without AF | 0/140 (0.0)
Lee [25]	Prospective cohort	374	63 (20–89)	254 (68)	Stroke | 45/374 (12.0)
Ajlan [26]	Retrospective cohort	47	52 (11)	25 (53)	Stroke of suspected cardioembolic source | 2/47 (4.3)
Taina [27]	Prospective cohort	102	62 (11)	70 (69)	Stroke or TIA of suspected cardioembolic source without AF | 0/102 (0.0)
Yeo [8]	Prospective open-pilot	20	64 (12)	13 (65)	Stroke patients eligible for reperfusion therapy | 4/20 (20.0)

*AF* atrial fibrillation, *NR* not reported, *Stroke* ischemic stroke, *TIA* transient ischemic attack

^*^The study was divided into three different periods/groups. Only the period/group in which patients were assessed with both echocardiography and CT is included in this table

Eleven of 14 studies were conducted exclusively in ischemic stroke patients, while three studies also included TIA patients. Four studies had a general ischemic stroke population regardless of medical history or stroke etiology, seven studies included ischemic stroke patients with a suspected cardioembolic source, two studies included patients with a suspected cardioembolic source other than AF, and one study excluded patients with a probable cardioembolic source. Overall, 308/1532 (20.1%) of patients for which this data were available had AF.

Details on CTA and echocardiography methods are reported in Table 2. Ten studies compared performance of CTA to TEE, four studies compared CTA to TTE. The order of tests was reported in 9/14 (64.3%) studies. Four studies performed cardiac CTA first, four echocardiography first, and in one study the order of tests varied. Time interval between stroke onset and first test was reported in 8/14 (57.1%) studies and was within 2 weeks for most of these studies. Time interval between tests was reported in 13/14 (92.8%) studies and ranged between 0 and 199 days. In most studies, the second test was done within 1 week after the first, four studies reported a time interval of approximately 1 day. Only one study performed cardiac CTA in the acute phase of ischemic stroke [8], i.e. within the time window for acute reperfusion therapy (intravenous thrombolysis and/or endovascular treatment). This study performed non ECG-gated CTA of the entire heart–brain axis in acute ischemic stroke patients within 4.5 h of symptom onset. For cardiac CTA, prospective ECG gating (when the scanner is triggered to scan the heart only during a predetermined phase of the cardiac cycle) was used in three studies, retrospective ECG gating (when all phases of the heart are scanned and images of the phase of interest are selected retrospectively) was used in eight studies, one study used both techniques and two studies did not use ECG registration. Nine studies used 64-slice CT or newer generation scanners, two studies used dual-source scanners.

**Table 2:** CT and echocardiography methods per study

References	First test: time interval between stroke and first test in days	Reported time interval between tests in days	TEE/TTE: MHz	CT type slice	Cardiac CTA ECG gating technique
Helgason [15]	CT: ≤ 21	< 1	TTE: NR	Imatron C-100	Retrospective
Love [16]	CT: range 0–13	Mean 3.5 (range 0–7)	TTE: 2.5/3.5	Imatron C-100	No ECG gating
Hur [17]	TEE: < 14	< 7	TEE: 5	64 s	Retrospective
Hur [18]	TEE: < 7	Mean 5 (SD 2.8)	TEE: 5	64 s	Retrospective
Boussel [19]	NR	TTE: mean 0.8 (SD 2.4) TEE: mean 2.5 (SD 2.8)	TTE: 2.5–3.5 TEE: 5	40 s	Retrospective
Kim [20]	NR	< 7	TEE: 7	64 s	Prospective
Ko [21]	NR	Median 4	TTE: 3.5 TEE: 7	64 s	Retrospective
Hur [22]	TEE: mean 6.8 (range 5–13)	Mean 2.3	TEE: 5–7	128 s dual source	Prospective
Kim [23]	NR	NR	TTE: 2.5	64 s	Retrospective
Sipola [24]	CT: mean 6 (SD 4)	< 1	TTE: 3 TEE: 6	16 s and 64 s	Retrospective
Lee [25]	TEE: < 7	Mean 4.64	TEE: 5–7	64 s and 128 s	Prospective
Ajlan [26]	TTE/CT: NR	Mean 19 (range 0–199)*	TTE: (NR)	128 s dual source	Prospective + retrospective
Taina [27]	NR	Mean 0.92 (SD 4.45)	TEE: 5–7	16 s	Retrospective
Yeo [8]	CT: < 1	< 1	TTE: 2.5–3.5 TEE: 5	64 s	No ECG gating

*AF* atrial fibrillation, *CTA* computed tomography angiography, *MHz* megahertz, *NR* data not reported, *SD* standard deviation, *TEE* transesophageal echocardiography, *TIA* transient Ischemic Attack, *TTE* transthoracic echocardiography

^*^Based on data from 34/47 patients

### Diagnostic yield

Data on detection of thrombi are provided in Table 3 (CTA versus TEE) and Table 4 (CTA versus TTE). In 1385 patients who underwent CTA and TEE, CTA detected cardiac thrombi in 87 (6.3%) patients compared to in 68 (4.9%) patients on TEE (*p* < 0.001). In a total of 22/1385 (1.6%) patients, thrombi were detected on CTA but not on TEE, while in 3/1385 (0.2%) patients thrombi were detected on TEE but not on CTA. Location of cardiac thrombi detected on CTA was LAA in 68/87 (78.2%), LA in 2/87 (2.3%), left ventricle in 3/87 (3.4%) and not specified in 14/87 (16.1%) patients. Location of cardiac thrombi detected on TEE was LAA in 56/68 (82.4%), LA in 4/68 (5.9%), left ventricle in 1/68 (1.5%) and not specified in 7/68 (10.3%) patients.

**Table 3:** Patients with cardiac thrombi detected on CT angiography (CTA) versus transesophageal echocardiography (TEE)

References	*N*	Patients with thrombi on CTA	Patients with thrombi on TEE	Patients with thrombi on CTA but not on TEE	Patients with thrombi on TEE but not on CTA
Hur [17]	101	12	8	4	0
Hur [18]	137	12	12	0	0
Boussel [19]	39	1	0	1	0
Kim [20]	314	29	23	6	0
Ko [21]	75	8	1	7	0
Hur [22]	83	13	13	0	0
Sipola [24]	140	1	3	1	3
Lee [25]	374	6	6	0	0
Taina [27]	102	3	0	3	0
Yeo [8]	20	2	2	0	0
Total	1385	87 (6.3%)	68 (4.9%)	22 (1.6%)	3 (0.2%)

*N* indicates the number of patients with ischemic stroke who underwent both CTA and TEE

**Table 4:** Patients with cardiac thrombi detected on CT angiography (CTA) versus transthoracic echocardiography (TTE)

References	*N*	Patients with thrombi on CTA	Patients with thrombi on TTE	Patients with thrombi on CTA but not on TTE	Patients with thrombi on TTE but not on CTA
Helgason [15]	38	11	2	11*	2
Love [16]	36	6	10	1	5
Kim [23]	62	0	0	0	0
Ajlan [26]	47	6	0	6	0
Total	183	23 (12.5%)	12 (6.6%)	18 (9.8%)	7 (3.8%)

*N* indicates the number of patients with ischemic stroke who underwent both CTA and TTE

^*^In this study CTA detected two thrombi in one patient

In 183 patients who underwent CTA and TTE, CTA detected thrombi in 24 (13.1%) patients compared to in 12 (6.6%) on TTE (*p* = 0.010). In 18/183 (9.8%) patients, thrombi were detected on CTA but not on TTE, in 7/183 (3.8%) patients thrombi were detected on TTE but not on CTA. Location of cardiac thrombi detected on CTA was LAA in 2/24 (8.3%), LA in 9/24 (37.5%), left ventricle in 7/24 (29.2%) and not specified in 6/24 (25.0%) patients. Location of cardiac thrombi detected on TTE was LA in 2/12 (16.7%) and not specified in 10/12 (83.3%).

### Diagnostic accuracy

**Fig. 2:**
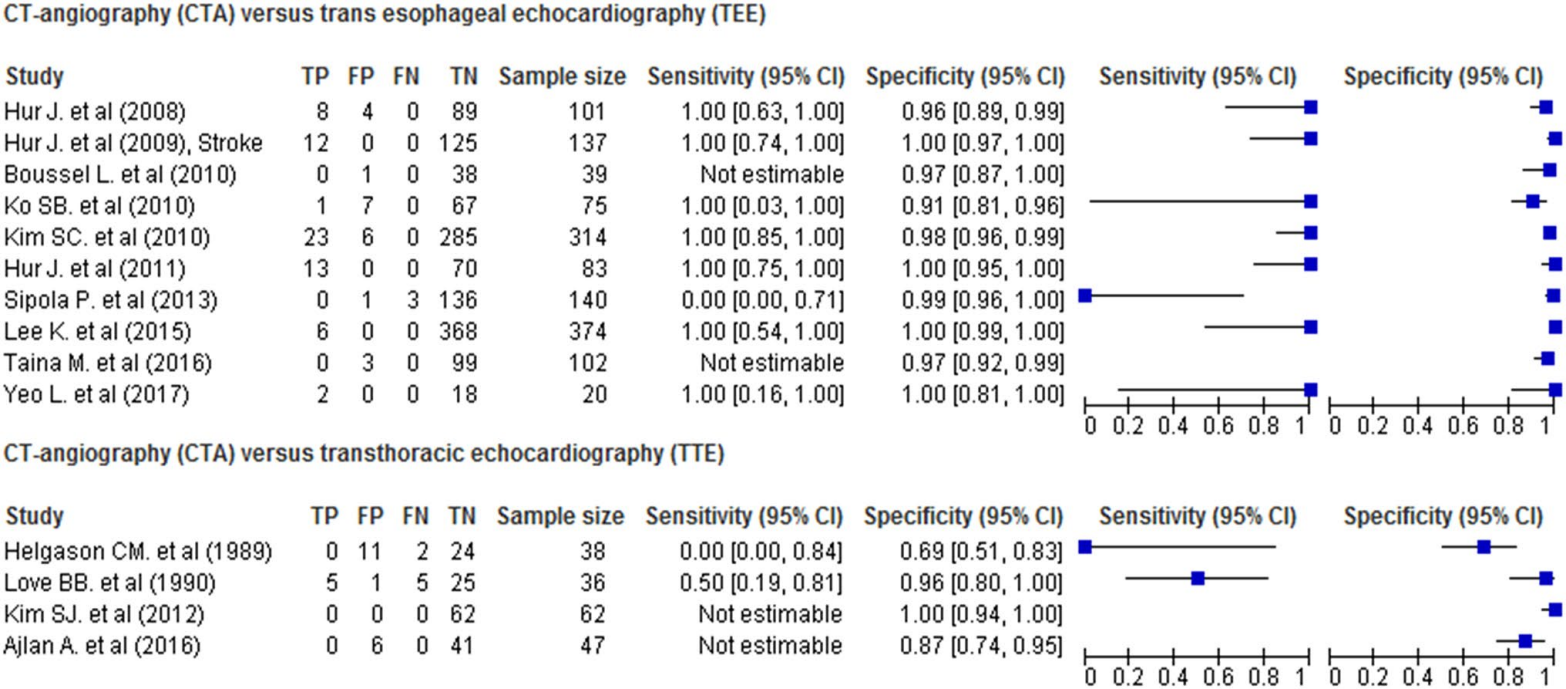
Sensitivity and specificity of cardiac CT angiography compared to echocardiography for detection of cardiac thrombus in stroke patients. *FN* false negative, *FP* false positive, *TN* true negative, *TP* true positive

Sensitivity of cardiac CTA with reference standard TEE was estimable in 8/10 studies and was 100% in 7/8 and 0% in 1/8 studies (Fig. 2). Specificity was calculated in 10/10 TEE studies and ranged between 91 and 100%. The pooled sensitivity of CTA compared to TEE was 86.0% (95% CI 65.6–95.2%) and the pooled specificity was 97.4% (95% CI 95.0–98.7%) (Online Resource Figure IIa).

Sensitivity of cardiac CTA with reference standard TTE was estimable in 2/4 studies and was 0% and 50%, respectively. Specificity was calculated in 4/4 TTE studies and ranged between 69 and 100%. The pooled sensitivity of CTA compared to TTE was 37.0% (95% CI 12.6–70.4%), the pooled specificity was 83.9% (95% CI 63.4–94.0%) (Online Resource Figure IIb).

The pooled sensitivity of CTA compared to TEE in the 7/10 CTA studies which used 64-slice CT scanners or newer generations was 93.7% (95% CI 82.6–97.9%) and the pooled specificity was 96.7% (95% CI 93.8–99.2%). After excluding two studies which used outdated CT scanners, there were insufficient data to perform a pooled analysis for sensitivity and specificity of CTA versus TTE.

The pooled sensitivity of CTA compared to TEE in the 4/10 studies in which the time interval between the two investigations was approximately 1 day was 47.7% (95% CI 7.7–90.9%) and the pooled specificity was 97.6% (95% CI 93.4–99.1%). After excluding two studies with a time interval exceeding 1 day and one with an unreported time interval, there were insufficient data to perform a pooled analysis for sensitivity and specificity of CTA versus TTE.

## Discussion

In our systematic review, we used data of 1568 ischemic stroke patients to determine the diagnostic yield of CTA and echocardiography for detection of cardiac thrombi. Our results suggest that cardiac thrombi are more often detected on cardiac CTA than on TEE or TTE. When examined in terms of diagnostic accuracy, CTA appears to have high sensitivity and specificity compared to TEE, while there was insufficient data for conclusions on sensitivity and specificity for CTA versus TTE. A previous meta-analysis found that cardiac CTA is a reliable alternative to TEE for detection of left atrium and left atrial appendage thrombi [28]. Our study distinguishes itself by focusing specifically on the ischemic stroke/TIA population, reporting also on left ventricular thrombi and the diagnostic yield of CTA versus TTE, and incorporating recent studies.

The methodological quality of studies was low in many aspects. Only 2/14 studies had a low risk of bias on all domains of the QUADAS-2 checklist [16, 18]. In addition, the definition of cardiac thrombus on CTA and echocardiography was often not reported and varied, which could have led to reporting bias. Furthermore, study populations were quite heterogeneous, which could have influenced the results. As expected, a larger proportion of cardiac thrombi was found in study populations with suspected cardioembolic source, followed by general ischemic stroke populations. In the study of Kim et al. [23], patients with AF or any history of cardiac disease were excluded and no cardiac thrombi were found, despite the fact that this study contained the largest patient population for studies comparing CTA to TTE.

Time interval between stroke and first test was not reported in almost half of the included studies. Also, studies that did report the interval had an interval of up to 7 days, which may be too late to detect thrombi, as there is evidence that cardiac thrombi may vanish within time, especially after administration of intravenous thrombolysis [29].

Four studies reported a time interval between tests of approximately 1 day [8, 15, 24, 27]. Of these studies, only one (*N* = 20) performed CTA on the same day as the ischemic stroke event and in this study CTA and TTE detected an equal amount of cardiac thrombi [8]. A minimal time interval between tests is important when comparing the two modalities, as especially the diagnostic yield of the second test that is performed can be negatively influenced due to this delay. In our systematic review, half of the studies performed echocardiography first and half CTA first.

Ambiguity remains regarding the additional value of ECG-gated imaging. Non-ECG gating is easier to implement and requires less expertise, however, ECG gating improves image quality of the moving heart. In this study, direct comparison of ECG gating versus non ECG gating was not possible due to the heterogeneity in study design, patient population and CT scanners. This also applies to the value of prospective versus retrospective ECG gating. Prospective ECG gating (or ECG triggering) allows for imaging of the heart in a specific point in the cardiac cycle (e.g. diastole, when the cardiac chambers can be optimally visualized) and results in less exposure to radiation than retrospective ECG gating. However, retrospective ECG gating (imaging of the heart throughout the cardiac cycle and selecting the desired images retrospectively) may increase the chance to acquire high-quality images in patients with AF or other heart rhythm irregularities.

Overall, in our analysis CTA detected more thrombi than echocardiography and CTA had high sensitivity and specificity compared to TEE. Not surprisingly, the pooled sensitivity of CTA compared to TEE further increased when outdated scanners were excluded from the analysis. However, due to the great heterogeneity between studies, interpretation of the value of these results for everyday practice is difficult and should be done with caution. Studies that used older CT scanners, did not define thrombus on CTA, or performed CTA as second test are more likely to present false positive results. On the other hand, studies that used modern CT scanners, defined thrombus on CTA and echocardiography and performed CTA first are more likely to present true positive results. Interestingly, pooled sensitivity of CTA compared to TTE was low. This is possibly due to the fact that there was no patient level agreement on the thrombi that were found with both modalities. CTA did find more thrombi in total, but in some other patients TTE detected thrombi that were not found on CTA.

A recent review on cardiac CTA and TEE offers helpful advice and illustrative images on which technique to use in certain clinical settings [30], including cryptogenic stroke. A complicating factor is that criteria for cryptogenic stroke are not standardized. It may be worthwhile to study the diagnostic yield of cardiac CTA versus TEE in embolic stroke of undetermined source (ESUS). Per ESUS definition [3] CTA and TEE would be add-ons to TTE in this scenario. Notably, the recommendations in the review [30] are based in part on the aforementioned meta-analysis [28] of studies published before CTA from aortic arch to intracranial vessels became routine care for patient selection for endovascular treatment in most stroke centers. The review does not discuss the potential role of cardiac CTA as part of the acute phase work-up. A challenge in this context is that for effective implementation of heart–brain axis CTA in the acute phase of ischemic stroke in clinical practice it is paramount to establish interdisciplinary agreements for the responsibility and time frame for systematic reporting of the widened field, fitting with local preferences.

Although CTA may be a promising alternative to echocardiography for detection of cardiac thrombi and other high-risk cardio-aortic sources of embolism in ischemic stroke patients, it is unlikely CTA will fully replace echocardiography. Limitations of CTA include lack of dynamic heart function data, exposure to radiation and contrast media, suboptimal image quality in patients with tachycardia or arrhythmias and unsuitability as a bedside technique. Adjustment of the CTA protocol to include dynamic data is possible using retrospective ECG gating with cine sequences, but it results in increased exposure to radiation. Even though additional radiation associated with cardiac CTA is low if modern CT scanners are used [11], it remains a disadvantage particularly for young patients. Small studies have demonstrated it is feasible to perform non-ECG-gated heart–brain axis CTA in the acute phase of ischemic stroke quickly and without additional contrast media [8–10]. However, it is likely that ECG gating and cardiac contrast injection protocols increase image quality and diagnostic yield, resulting in longer acquisition times and increased risk of renal injury.

Though outside the scope of our study, a third technique with growing potential for imaging of the heart in stroke is magnetic resonance imaging (MRI) [31, 32]. Cardiac MRI is non-invasive and can provide functional and structural information. Main disadvantages include relatively high cost, less availability (especially in the acute phase of ischemic stroke), long acquisition times and lower spatial resolution compared with CTA.

The main limitation of this paper is the great heterogeneity between studies, missing data, and low quality of the included studies. Second, choosing diagnostic yield as our primary endpoint makes interpretation of disagreements in results between CTA and echocardiography challenging. This interpretation depends on factors such as the order in which tests were performed, time interval between tests and the type of CT scanner which was used. However, we believe that due to the lack of a true gold standard, presenting results in terms of sensitivity/specificity as primary endpoint might not do justice to the value of CTA, especially in studies in which echocardiography is more likely to present false negative results.

## Conclusion

On the basis of our systematic review, CTA may be an attractive alternative to echocardiography for detection of cardiac thrombi in ischemic stroke patients. Heterogeneity between studies and the lack of an optimal gold standard complicate interpretation of diagnostic accuracy. Now that CTA of the cervical and intracranial arteries has become routine care to determine eligibility for endovascular treatment and additional radiation as a result of extending the CTA to include the heart is low with modern CT scanners, large multicenter prospective studies focusing on the diagnostic yield of cardiac CTA in the acute phase of ischemic stroke versus echocardiography (TTE and/or TEE) for detection of cardiac thrombi (and other high-risk cardio-aortic sources of embolism) are warranted. Another context worthwhile of study would be the diagnostic yield of cardiac CTA versus TEE in patients with ESUS.

## Electronic supplementary material

Below is the link to the electronic supplementary material.

Supplementary file1 (PDF 212 kb)

Supplementary file2 (PDF 138 kb)
