# Correction to: CT angiography vs echocardiography for detection of cardiac thrombi in ischemic stroke: a systematic review and meta-analysis

**DOI:** 10.1007/s00415-020-10126-9

**Published:** 2020-08-25

**Authors:** Nina-Suzanne Groeneveld, Valeria Guglielmi, Mariska M. G. Leeflang, S. Matthijs Boekholdt, R. Nils Planken, Yvo B. W. E. M. Roos, Charles B. L. M. Majoie, Jonathan M. Coutinho

**Affiliations:** 1 Department of Neurology, Amsterdam UMC, University of Amsterdam, Amsterdam, The Netherlands; 2 Department of Clinical Epidemiology and Biostatistics and Bioinformatics Amsterdam Public Health, Amsterdam UMC, University of Amsterdam, Amsterdam, The Netherlands; 3 Department of Cardiology, Amsterdam UMC, University of Amsterdam, Amsterdam, The Netherlands; 4 Department of Radiology and Nuclear Medicine, Amsterdam UMC, University of Amsterdam, Amsterdam, The Netherlands

## Correction to: Journal of Neurology (2020) 267:1793–1801 10.1007/s00415-020-09766-8

The original version of this article unfortunately contained a mistake. In the author list, the first and last names of two authors, S. Matthijs Boekholdt and R. Nils Planken, were tagged incorrectly. Therefore, author names are abbreviated wrongly in Springerlink. The first and last names should be as follows:

First name: S. Matthijs

Last name: Boekholdt

First name: R. Nils

Last name: Planken

