# Supplementary material for: CT angiography vs echocardiography for detection of cardiac thrombi in ischemic stroke: a systematic review and meta-analysis
Source: J Neurol. 2020 Mar 5;267(6):1793–801. doi: 10.1007/s00415-020-09766-8 (PMC7293690; doi:10.1007/s00415-020-09766-8)
Supplement: Supplementary file 1 — Supplementary file1 (PDF 212 kb) [file 415_2020_9766_MOESM1_ESM.pdf]

## Online Resource

CT-angiography vs echography for detection of cardiac thrombi in ischemic stroke: a systematic review and meta-analysis

Journal of Neurology

Nina-Suzanne Groeneveld*, Valeria Guglielmi*, Mariska M.G. Leeflang, S. Matthijs Boekholdt, R. Nils Planken, Yvo B.W.E.M. Roos, Charles B.L.M. Majoie, Jonathan M. Coutinho

*Joint first authorship

Correspondence: Jonathan M. Coutinho, Department of Neurology, Amsterdam UMC, University of Amsterdam, Meibergdreef 9, 1105 AZ Amsterdam, The Netherlands. E-mail: j.coutinho@amsterdamumc.nl

### Supplemental Tables

Table I Literature search strategy

Table IIa Criteria used to assess methodological quality of included studies: risk of bias

Table IIb Criteria used to assess methodological quality of included studies: concerns regarding applicability

### Supplemental Figures

Figure Ia Methodological quality of included studies according to QUADAS-2 checklist: risk of bias

Figure Ib Methodological quality of included studies according to QUADAS-2 checklist: concerns regarding applicability

Figure IIa Summary receiver operating characteristic (SROC) curve (bivariate model) for diagnostic accuracy of cardiac CT-angiography (CTA) compared to transesophageal echocardiography (TEE)

Figure IIb Summary receiver operating characteristic (SROC) curve (bivariate model) for diagnostic accuracy of cardiac CT-angiography (CTA) compared to transthoracic echocardiography (TTE)

### Other Supplemental Data

Reference list of excluded studies, appendix to Figure 1. Flowchart of study selection

**Table I** Literature search strategy

| **Ovid MEDLINE search** | | |
|---|---|---|
| **#** | **Searches** | **Results** |
| 1. | exp brain ischemia/ or stroke/ or exp brain infarction/ or hypoxia-ischemia, brain/ or exp "Intracranial Embolism and Thrombosis"/ or (stroke or cerebrovasc$ or brain vasc$ or cerebral vasc$ or cva$ or apoplex$ or isch?emi$ attack$).ti,ab. or ((brain$ or cerebr$ or cerebell$ or cortical or vertebrobasilar or lacunar or hemispher$ or intracran$ or intracerebral or infratentorial or supratentorial or MCA or anterior circulation or posterior circulation or basal ganglia) adj5 (isch?emi$ or infarct$ or thrombo$ or emboli$ or occlus$ or hypox$ or obstruction)).ti,ab. | 362297 |
| 2. | ((ecg or electrocardiograph* or appendage or atrial or ventricle or cardiac or heart) adj5 ("compute* tomograph*" or "compute* aided tomograph*" or CT or CCT)).ti,ab,kf. or ((heart/ or heart atria/ or atrial appendage/ or heart ventricles/) and Computed Tomography Angiography/) | 9381 |
| 3. | 1 and 2 | 502 |
| 4. | exp animals/ not humans/ | 4467985 |
| 5. | 3 not 4 | 530 |

| **Embase search** | | |
|---|---|---|
| **#** | **Searches** | **Results** |
| 1. | exp brain ischemia/ or exp cerebrovascular accident/ or exp brain infarction/ or exp hypoxic ischemic encephalopathy/ or exp occlusive cerebrovascular disease/ or ((brain$ or cerebr$ or cerebell$ or cortical or vertebrobasilar or lacunar or hemispher$ or intracran$ or intracerebral or infratentorial or supratentorial or MCA or anterior circulation or posterior circulation or basal ganglia) adj5 (isch?emi$ or infarct$ or thrombo$ or emboli$ or occlus$ or hypox$ or obstruction)).ti,ab. | 410576 |

| | | |
|---|---|---|
| 2. | ((ecg or electrocardiograph* or appendage or atrial or ventricle or cardiac or heart) adj5 ("compute* tomograph*" or "compute* aided tomograph*" or CT or CCT)).ti,ab,kw. or ((heart/ or heart atrium/ or heart ventricle/) and computed tomographic angiography/) | 16766 |
| 3. | 1 and 2 | 972 |
| 4. | (exp experimental organism/ or animal tissue/ or animal cell/ or exp animal disease/ or exp carnivore disease/ or exp bird/ or exp experimental animal welfare/ or exp animal husbandry/ or animal behavior/ or exp animal cell culture/ or exp mammalian disease/ or exp mammal/ or exp marine species/ or nonhuman/ or animal.hw.) not human/ | 6848224 |
| 5. | 3 not 4 | 966 |
| 6. | (embase or elsevier or canadian).cr. | 25384379 |
| 7. | 5 and 6 | 1000 |

**Table IIa** Criteria* used to assess methodological quality of included studies: risk of bias

| Quality item | Positive score (low risk of bias) |
|---|---|
| Domain 1: Patient selection | |
| 1. Was a consecutive or random sample of patients enrolled? | The period of inclusion and way of including patients was clearly described and consecutive ischemic stroke patients were included. |
| 2. Was a case-control design avoided? | A case-control design - in which two existing groups differing in outcome are identified and compared on the basis of some supposed causal attribute - was avoided. |
| 3. Did the study avoid inappropriate exclusions? | Withdrawals of the study were explained and red flags regarding inclusion of patients were avoided ('excluding difficult to diagnose patients'). |
| 4. Were selection criteria clearly described? | It was clear how and which ischemic stroke patients were selected to undergo cardiac CTA and echocardiography. |
| Domain 2: Index test | |
| 5. Were the index test results interpreted without knowledge of the results of the reference standard? | All reviewers of the index test results were blinded to the results of the reference standard. |
| 6. If a threshold was used, was it pre-specified? | The definition of cardiac thrombus was provided. |
| 7. Was the execution of the index test described in sufficient detail to permit replication of the test? | The CT scan protocol (scanner type, acquisition mode, reconstruction method, scanning direction, tube current, tube voltage, contrast load, ECG-gating) was described. |
| Domain 3: Reference standard | |
| 8. Is the reference standard likely to correctly classify the target condition? | TTE or TEE was used as a reference standard. |
| 9. Were the reference standard results interpreted without knowledge of the results of the index test? | All reviewers of the reference standard results were blinded to the results of the index test. |
| 10. Was the execution of the reference test described in sufficient detail to permit replication of the test? | The echocardiography protocol (type, probes (MHz), different views, patient position) was described. |
| Domain 4: Flow and timing | |

| | |
|---|---|
| 11. Was there an appropriate interval between index test(s) (CT) and reference standard (echocardiography)? | Mean/median time period between cardiac CTA and echocardiography was ≤ 7 days. |
| 12. Did all patients receive a reference standard? | All patients included underwent echocardiography. |
| 13. Did patients receive the same reference standard? | All patients included underwent the same reference standard (TTE or TEE). |
| 14. Were all patients included in the analysis? | All patients who underwent both investigations were also included in the final analysis. |

CTA indicates computed Tomography Angiography; ECG, electrocardiogram; MHz, Megahertz; TEE, trans esophageal echocardiography; TTE, transthoracic echocardiography;
* Based on Quality Assessment of Diagnostic Accuracy Studies-2 (QUADAS-2) checklist.

**Table IIb** Criteria* used to assess methodological quality of included studies: concerns regarding applicability

| Quality item | Positive score (low concern) |
|---|---|
| 1. Is there concern that the spectrum of patients is not representative of the patients who will receive the test in practice? | Patients with ischemic stroke were included, with or without AF and with or without other cardiac medical history. |
| 2. Is there concern that the index test, its conduct, or interpretation differ from the review question? | CT-scanner type and method of assessing images was acceptable and clearly described. |
| 3. Is there concern that the target condition as defined by the reference standard does not match the review question? | Type of echo probe and method of assessing images was acceptable and clearly described. |

AF indicates atrial fibrillation; CT, computed tomography.
* Based on Quality Assessment of Diagnostic Accuracy Studies-2 (QUADAS-2) checklist.

**Figure Ia** Methodological quality of included studies according to QUADAS-2 checklist: risk of bias

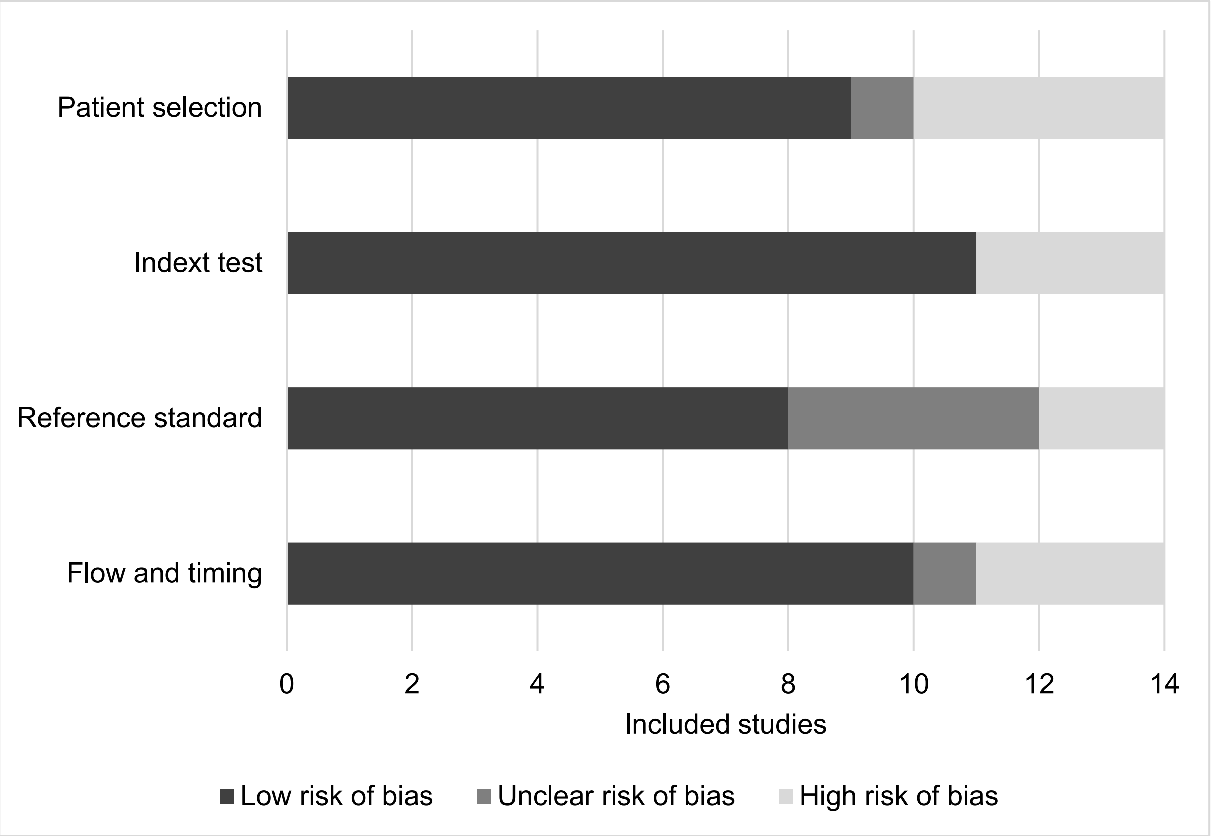


QUADAS indicates Quality Assessment of Diagnostic Accuracy Studies.

**Figure Ib** Methodological quality of included studies according to QUADAS-2 checklist: concerns regarding applicability

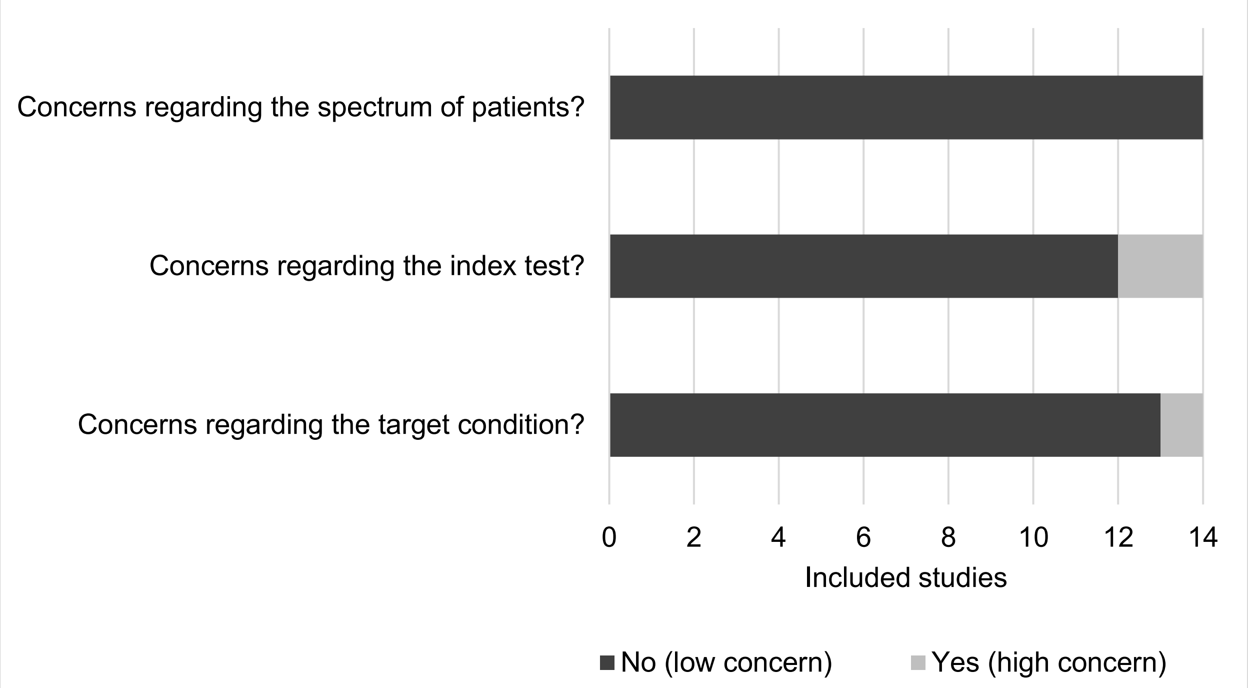


QUADAS indicates Quality Assessment of Diagnostic Accuracy Studies.

**Figure IIa** Summary receiver operating characteristic (SROC) curve (bivariate model) for diagnostic accuracy of cardiac CT-angiography (CTA) compared to transesophageal echocardiography (TEE)*

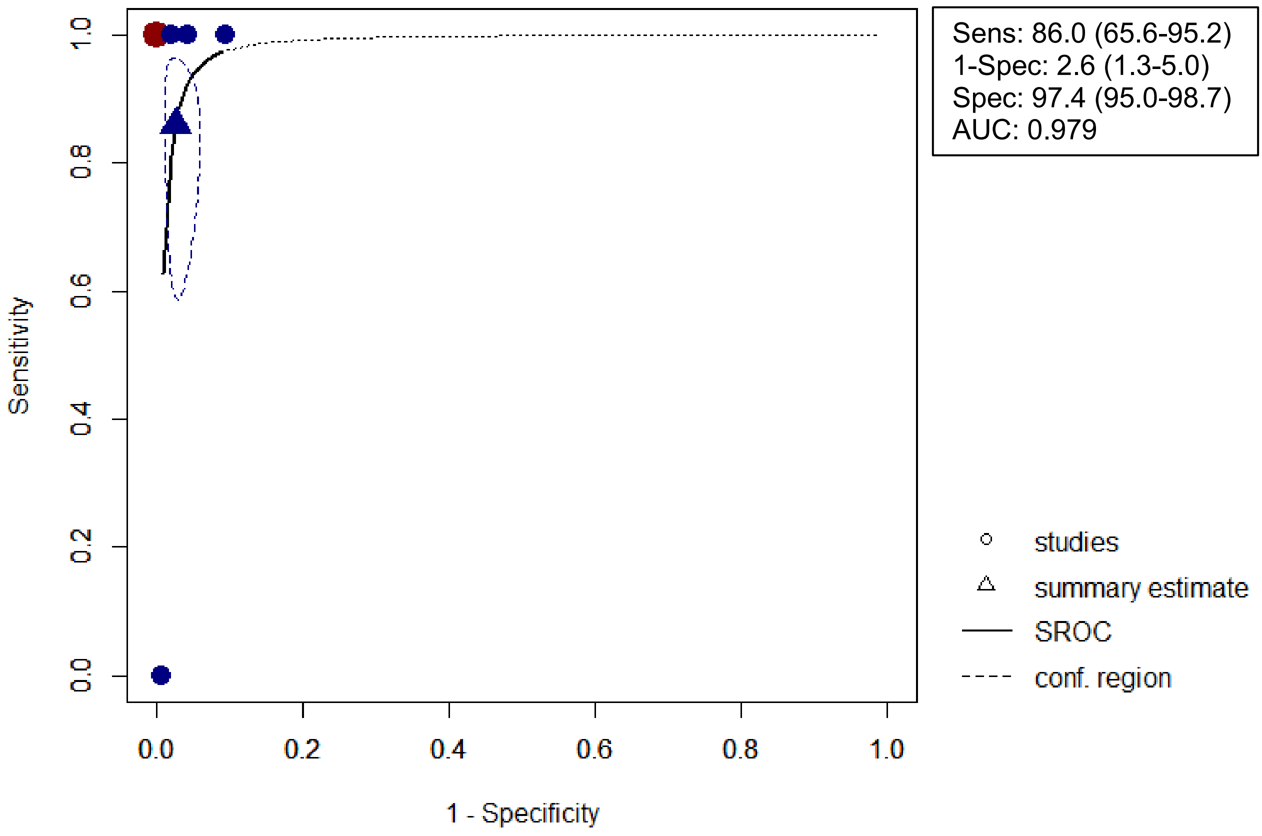


SROC indicates Summary receiver operating characteristic; AUC, area under the curve; sens, sensitivity; spec, specificity.
*The red dot indicates 4 studies with similar sensitivity of 100.

**Figure IIb** Summary receiver operating characteristic (SROC) curve (bivariate model) for diagnostic accuracy of cardiac CT-angiography (CTA) compared to transthoracic echocardiography (TTE)

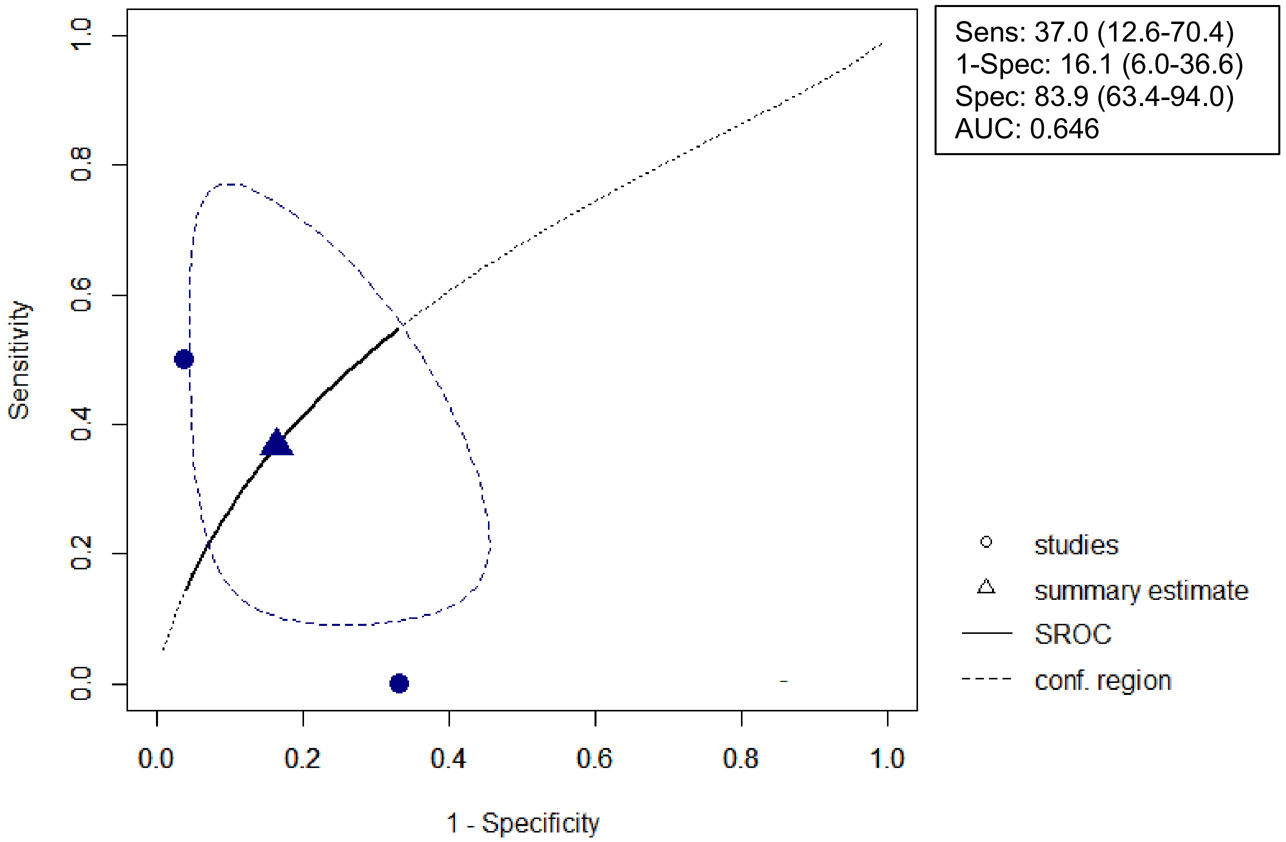


SROC indicates Summary receiver operating characteristic; AUC, area under the curve; sens, sensitivity; spec, specificity.

**Reference list of excluded studies, appendix to Figure 1. Flowchart of study selection**


Conference abstract with insufficient information (1-12)

1. Uchiyama S, Yamazaki M, Iwata M, Maruyama S (1996) Diagnosis of intracardiac thrombi by various imaging techniques and activation of platelets and coagulation-fibrinolysis in patients with cardioembolic stroke. Rinsho Shinkeigaku 36:429-435
2. Desai JA, Dobson JL, Salahudeen S, Flood J, Nolan RL, Jin AY (2011) Cardiac MRI for the detection of proximal sources of embolism in stroke and TIA patients. Canadian Journal of Neurological Sciences 1:S75
3. Desai JA, Flood JR, Dobson J, Salahudeen SR, Nolan RL, Jin AY (2011) Cardiac MRI findings in the etiological workup of stroke and TIA. Stroke 42:e594
4. Knot J, Petr R, Linkova H, Daniel J, Labos M, Widimsky P (2012) Detection of left atrial appendage thrombi using multidetector CT-angiography compared with transesophageal echocardiography. European Heart Journal 1:87
5. Konoeda F, Dembo T, Yamada S, Itoh Y, Jinzaki M, Suzuki N (2012) Utility of 64-section multi-detected cardiac CT in detection of thrombus and circulation in left atrium appendage: Comparison with trans-oesophageal echocardiography. European Journal of Neurology 19:156
6. Kelliher E, Galvin L, Power S, Pearly-Ti J, Thornton J, Brennan P, Looby S (2012) Cardiac and aortic arch findings on CT angiography head and neck studies. Neuroradiology 1:S171-S172
7. Manchev L, Toneva J, Mitev M, Milanova V, Zafirova E, Manolova T, Manchev I (2013) Computed tomographic, electrocardiographic and clinical investigations in patients with ischemic strokes. Rentgenologiya i Radiologiya 52:115-119
8. Ryoo S, Moon GJ, Lee CB, Kim SJ, Chung CS, Lee KH, Bang OY (2013) Determinants for cryptogenic stroke: Clinico-radiologic findings. Stroke:44
9. Kim GM, Helenius J, Arsava EM, Ay H (2014) Testing the utility of an emergency room-based stroke evaluation protocol. Stroke:45
10. Holswilder G, Wermer M, Kroft L, De Roos A, Holman E, Kruyt N, Van Walderveen M (2018) Value of cardiac CT-angiography in the diagnostic workup of patients with TIA and acute ischaemic stroke: Prevalence of cardioembolic risk sources and therapeutic implications. European Stroke Journal 3:34
11. Parikh A, Dodds J (2018) Estimated ejection fraction in patients with acute ischemic stroke and left ventricular thrombi. European Stroke Journal 3:315
12. Parikh A, Dodds J (2018) Length-of-stay and demographics in acute ischemic stroke with left atrial or left ventricular thrombus. European Stroke Journal 3:406

Did not allow data extraction on cardiac thrombus cases (13-16)

13. Kim JT, Yoo SH, Kwon JH, Kwon SU, Kim JS (2006) Subtyping of ischemic stroke based on vascular imaging: analysis of 1,167 acute, consecutive patients. Journal of clinical neurology (Seoul, Korea) 2:225-230
14. Hussain SI, Gilkeson RC, Suarez JI, Tarr R, Schluchter M, Landis DM, Zaidat OO (2008) Comparing multislice electrocardiogram-gated spiral computerized tomography and transesophageal echocardiography in evaluating aortic atheroma in patients with acute ischemic stroke. Journal of stroke and cerebrovascular diseases : the official journal of National Stroke Association 17:134-140

15. Kim YJ, Hur J, Shim CY, Lee HJ, Ha JW, Choe KO, Heo JH, Choi EY, Choi BW (2009) Patent foramen ovale: diagnosis with multidetector CT--comparison with transesophageal echocardiography. Radiology 250:61-67
16. Dodds J, Parikh A (2018) Affected vascular territories in presumed cardioembolic stroke in patients with left atrial or ventricular thrombi. European Stroke Journal 3:467

Did not involve ischemic stroke or TIA patients (17-21)

17. Allard-Latour G, Schlama S, Aubran M, Trigano JA, Juhan C, Torresani J (1985) Archives des Maladies du Coeur et des Vaisseaux 78:1249-1254
18. Foster CJ, Sekiya T, Love HG, Brownlee WC, Griffin JF, Isherwood I (1987) Identification of intracardiac thrombus: comparison of computed tomography and cross-sectional echocardiography. The British journal of radiology 60:327-33
19. Feuchtner GM, Dichtl W, Bonatti JO, Jodocy D, Muller S, Hintringer F, Gradl J, Klauser A, Cury RC (2008) Diagnostic accuracy of cardiac 64-slice computed tomography in detecting atrial thrombi. Comparative study with transesophageal echocardiography and cardiac surgery. Investigative radiology 43:794-801
20. Homsi R, Nath B, Luetkens JA, Schwab JO, Schild HH, Naehle CP (2016) Can Contrast-Enhanced Multi-Detector Computed Tomography Replace Transesophageal Echocardiography for the Detection of Thrombogenic Milieu and Thrombi in the Left Atrial Appendage: A Prospective Study with 124 Patients. RoFo : Fortschritte auf dem Gebiete der Rontgenstrahlen und der Nuklearmedizin 188:45-52
21. Hur J, Kim YJ, Lee HJ, Nam JE, Hong YJ, Kim HY, Lee JW, Choi BW (2012) Cardioembolic stroke: dual-energy cardiac CT for differentiation of left atrial appendage thrombus and circulatory stasis. Radiology 263:688-695

No reference standard (22-25)

22. Bogousslavsky J, Van Melle G, Regli F (1988) The Lausanne Stroke Registry: analysis of 1,000 consecutive patients with first stroke. Stroke 19:1083-1092
23. Mattioli AV, Castellani ET, Casali E (1994) [Transesophageal echocardiography in the assessment of patients with atrial fibrillation and stroke]. Cardiologia (Rome, Italy) 39:101-10
24. Furtado AD, Adraktas DD, Brasic N, Cheng SC, Ordovas K, Smith WS, Lewin MR, Chun K, Chien JD, Schaeffer S, Wintermark M (2010) The triple rule-out for acute ischemic stroke: imaging the brain, carotid arteries, aorta, and heart. AJNR American journal of neuroradiology 31:1290-1296
25. Iwasaki K, Matsumoto T, Kawada S (2016) Potential Utility of Multidetector Computed Tomography to Identify both Cardiac Embolic Sources and Coronary Artery Disease in Patients with Embolic Stroke. Cardiology 133:205-210

Did not have a RCT/ cross-sectional diagnostic accuracy study design (26-28)

26. Delcker A, Diener HC (1991) [Neurological diagnosis and therapeutic measures in cerebral embolism]. Herz 16:434-443
27. Kara K, Ozturk E, Sildiroglu O (2016) Role of Coronary CT Angiography in Patients with Stroke. Radiology 278:959
28. Kim JS, Park JW, Pak HN, Jang Y (2016) The novel application of intraprocedural cardiac computed tomography for left atrial appendage occlusion. European heart journal 37:1626

Overlap patient population (29)*

29. Hur J, Kim YJ, Lee HJ, Ha JW, Heo JH, Choi EY, Shim CY, Kim TH, Nam JE, Choe KO, Choi BW (2009) Left atrial appendage thrombi in stroke patients: detection with two-phase cardiac CT angiography versus transesophageal echocardiography. Radiology 251:683-690

* There was overlap in patient population with the following study: Hur J, Kim YJ, Lee HJ, Ha JW, Heo JH, Choi EY, Shim CY, Kim TH, Nam JE, Choe KO, Choi BW (2009) Cardiac computed tomographic angiography for detection of cardiac sources of embolism in stroke patients. Stroke 40:2073-2078
